# Reconciling Q waves and late gadolinium enhancement with no angiographic evidence of coronary disease: cardiac sarcoidosis presenting as decompensated heart failure

**Published:** 2010-06

**Authors:** RYAN P MORRISSEY,, KIRAN J PHILIP, ERNST R SCHWARZ

**Affiliations:** Cedars Sinai Heart Institute, Cedars-Sinai Medical Center, and David Geffen School of Medicine, University of California Los Angeles (UCLA), California, USA; Cedars Sinai Heart Institute, Cedars-Sinai Medical Center, and David Geffen School of Medicine, University of California Los Angeles (UCLA), California, USA; Cedars Sinai Heart Institute, Cedars-Sinai Medical Center, and David Geffen School of Medicine, University of California Los Angeles (UCLA), California, USA

**Keywords:** cardiac sarcoidosis, cardiac MRI (CMR), restrictive cardiomyopathy

## Abstract

Cardiac sarcoidosis is rare and subclinical involvement is four to five times more common than clinical involvement. Cardiac sarcoidosis is associated with a poor prognosis. ECG abnormalities are the most common presentation. However, as this case illustrates, it can also present as acute decompensated heart failure. Screening with cardiac positron emission tomography (PET) or magnetic resonance imaging (MRI) is highly suggested in patients with suspected disease. Diagnosis allows for early initiation of corticosteroids. Cardiac sarcoidosis is more common than previously thought. However, with treatment, survival may also be better than previously reported.

## Introduction

Sarcoidosis is a systemic granulomatous disease of unknown aetiology presenting in young and middle-aged adults and is almost three times more common in blacks than whites.^1^,^2^ The most common organ involved is the lung, but sarcoidosis can also affect multiple organ systems including the heart.^1^

Jonathan Hutchinson described the first case of cutaneous sarcoid in 1869.^3^ However, the illness was named by Cæsar Peter Møller Boeck in 1899.^4^ Bernstein et al. were the first to recognise cardiac involvement in 1929.^5^

Cardiac sarcoidosis most commonly manifests with abnormal ECG findings, including atrial and ventricular arrhythmias and varying degrees of atrio-ventricular block. Patients can present with symptoms of pre-syncope secondary to conduction abnormalities or symptoms of heart failure due to left ventricular dysfunction, mitral regurgitation or conduction abnormalities.^6^,^7^ Mortality is increased in sarcoidosis patients with cardiac involvement.^8^

Outcome data regarding conventional treatment of arrhythmias and heart failure in patients with cardiac sarcoidosis are lacking. Corticosteroid treatment improves mortality,^9^,^10^ and heart transplant recipients do as well as patients transplanted for other indications,^11^ although recurrence rates of cardiac sarcoidosis are not known.

Sarcoidosis should be in the differential for hilar lymphadenopathy, particularly in patients of African descent. This case illustrates that one should consider cardiac sarcoidosis and other infiltrative diseases when ECG or cardiac imaging findings do not reflect angiographic data.

## Case report

A 50-year-old African-American male presented to the emergency department with one week of shortness of breath at rest, which had prevented him from going to work, and five months of increasing dyspnoea on exertion. The patient denied any chest pain, palpitations, lower extremity swelling or cough. He had been experiencing night sweats for one week and endorsed a 10-lb (4.5-kg) weight loss over the previous five months. The patient, who worked in a warehouse, had been diagnosed in the past with reactive airway disease. He did not smoke but was formerly a heavy drinker and denied any history of illicit drug use. His family history was negative for heart and lung disease as well as malignancies.

On physical examination, temperature was 99.4° F (37.4° C), blood pressure was 115/68 mmHg, heart rate was 115 beats/ minute and regular, respiratory rate was 20 /minute, and oxygen saturation was 94% on two litres oxygen via nasal cannula. The patient was diaphoretic and respirations were augmented with accessory muscle use. The heart was tachycardic but with regular rhythm with occasional premature beats. S1 and S2 were normal without murmurs, rubs or gallops. The point of maximal impulse was not displaced. There was no jugular venous distention. Diffuse, coarse expiratory wheezes were present bilaterally without rales. Lower extremities were without oedema, and pulses were 2+ bilaterally. The digits of the hands exhibited clubbing. There were numerous hypopigmented plaques of 1 to 2 cm located on the upper back.

**Fig. 1.**
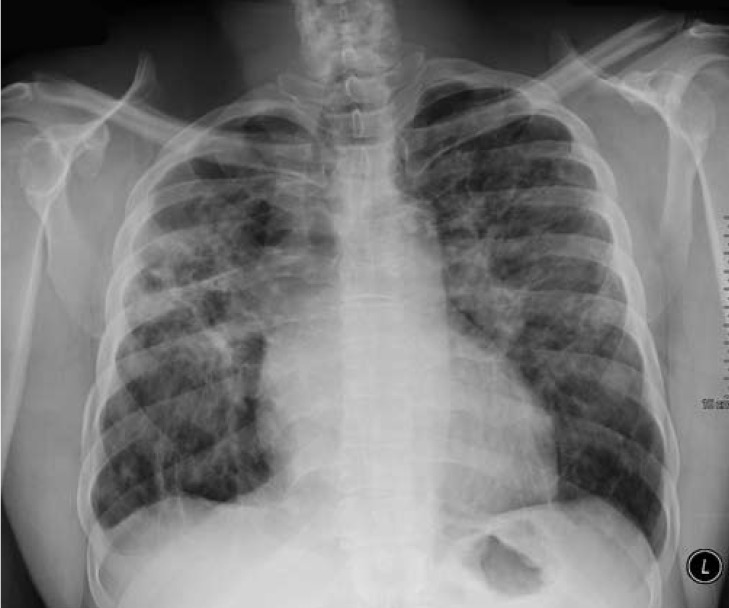
Plain film of the chest demonstrating extensive interstitial lung disease and peri-hilar consolidations versus increased pulmonary vasculature.

**Fig. 2.**
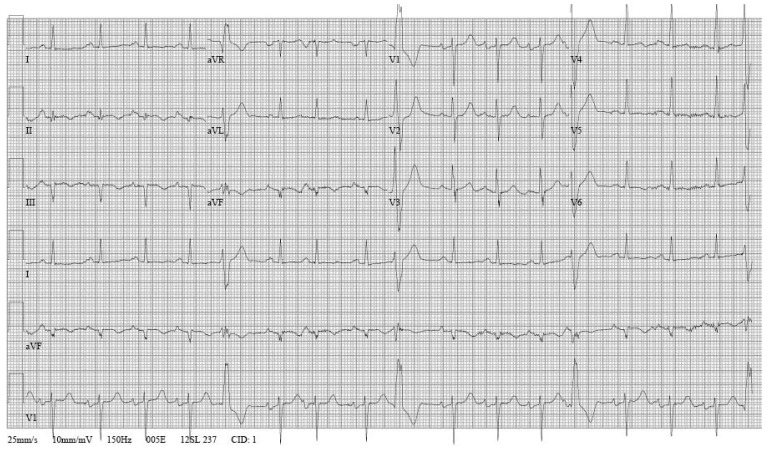
ECG demonstrating frequent PVCs (left ventricular in origin), non-specific atrio-ventricular delay, biatrial enlargement and evidence of an old inferior infarct.

An upright, two-view chest X-ray revealed bilateral upper lobe infiltrates and diffuse interstitial markings (Fig. 1). An ECG revealed normal sinus rhythm with several pre-ventricular contractions (PVCs) with right bundle branch morphologies (most likely sub-aortic valve, left ventricular outflow tract in origin),^12^ a ventricular rate of 100 beats/minute, non-specific atrio-ventricular delay, biatrial enlargement, and Q waves in leads II, III and aVF with inverted T waves (Fig. 2).

Laboratory evaluation was significant for mildly elevated AST at 151 U/l (normal < 46 U/l) and ALT at 70 U/l (normal < 53 U/l), LDH elevated at 438 U/l (normal < 260 U/l), and natriuretic peptide assay elevated at 460 pg/ml (normal < 100 pg/ ml). White blood cell count, haemoglobin and differential were all normal. Electrolytes including BUN and creatinine were also normal.

Overnight the patient required intubation for hypoxaemic respiratory failure. A bronchoscopy was performed which revealed bloody transudate. No infectious organism was identified in the blood or respiratory secretions, including fungus, acid-fast bacillus, Coccidiomycosis by serology or Pneumocystis carinii by direct fluorescent antibody. HIV 1 and 2 serology was negative.

A transthoracic two-dimensional echocardiogram revealed a dilated left ventricle with severe global left ventricular hypokinesis and an ejection fraction of 21%, without evidence of increased echogenicity compatible with infiltrative disease or fibrosis. E/E′ was not significantly elevated (12.7), pulmonary vein flow was diastolic-predominant and E/A was consistent with pseudo-normalisation (left ventricular diastolic dysfunction). There was moderate to severe mitral regurgitation, which was eccentric and posteriorly directed. Pulmonary artery systolic pressure was estimated at 19 mmHg; the inaccuracy of this estimate may have been due to positive-pressure ventilation, intrinsic lung disease, poor right ventricular function or inaccurate Doppler of the tricuspid regurgitation.

A high-resolution CT scan of the chest was performed which showed extensive bilateral parenchymal ground-glass opacities as well as focal areas of consolidation, bilateral hilar and mediastinal lymphadenopathy and multiple pulmonary nodules. Angiotensin converting enzyme level was 42 U/l (normal range 9–67 U/l).

Natriuretic peptide assay increased to 1 560 pg/ml. The patient was diuresed and eventually extubated. During his ICU stay the patient had several runs of non-sustained ventricular tachycardia.

Given the patient’s ECG, telemetry, echocardiographic and CT abnormalities as well as evidence of heart failure, there was a strong suspicion of cardiac sarcoidosis. Cardiac MRI (CMR) was obtained, which showed severe left ventricular dilatation (270 ml) and dysfunction (left ventricular ejection fraction of 22%) with dyskinesis of the inferior wall, akinesis of the lateral wall and septum, and hypokinesis of the apical and anterior walls.

**Fig. 3.**
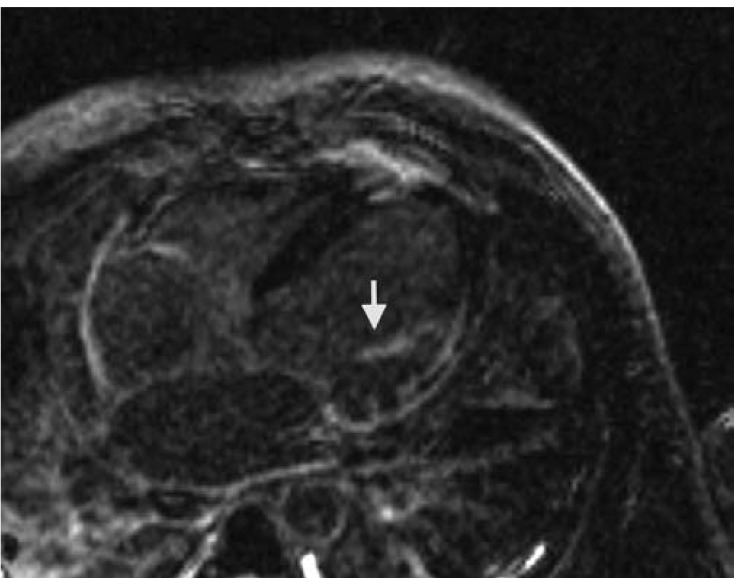
CMR demonstrating left ventricular wall thinning and delayed gadolinium enhancement of the papillary muscle (arrow).

Delayed gadolinium enhancement was seen inferolaterally (76–100%), laterally (51–75%), septally (26–50%), apically (26–50%) and anterolaterally (26–50%). There was at least moderate mitral regurgitation with both anterior and posterior papillary muscle delayed enhancement (Fig. 3). Delayed gadolinium enhancement was also seen in the distal right ventricular free wall and inferior wall. The transmural enhancement in the inferior and inferolateral walls and subendocardial enhancement in the distal anterior wall, septum and apex were thought to be most suggestive of prior infarctions involving the RCA and the distal LAD.

Right and left heart catheterisations were performed. The pulmonary pressure was 53/32 mmHg with a mean of 38 mmHg and a mean wedge pressure of 19 mmHg. Both the left and right coronary arteries were free of significant obstructive disease. Endomyocardial biopsy of the right ventricle was obtained, which showed moderate myofibre hypertrophy and increased interstitial fibrosis, but no evidence for active inflammation, myofibre degeneration, vasculitis, granulomas or neoplasia. Stains for iron and amyloid were negative.

**Fig. 4.**
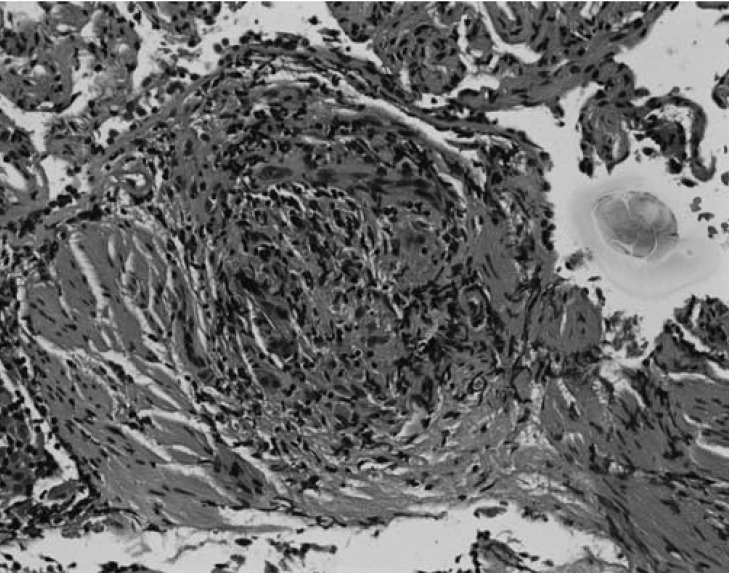
Transbronchial biopsy at high power showing a non-caseating granuloma.

Repeat bronchoscopy was performed with transbronchial biopsies of the right upper and middle lobes. The pathological examination revealed lung with mild chronic interstitial inflammation and bronchial wall with mild chronic inflammation and focal non-necrotising epithelioid granulomas. There was no evidence of tumour, viral changes or fungal elements (Fig. 4). A diagnosis of sarcoidosis was made and the patient was started on prednisone 30 mg per day in addition to his heart failure regimen (metoprolol succinate 50 mg per day, lisinopril 10 mg per day, spironolactone 25 mg per day, furosemide 20 mg per day and aspirin 81 mg per day).

He was doing well one year after diagnosis at clinical follow up, with subjective improvement in breathing and exercise capacity. His prednisone has been tapered to 30 mg every other day. He has thus far refused implantation of a cardiodefibrillator.

The patient’s ethnic background, and chest X-ray and chest CT findings were highly suggestive of sarcoidosis and, as will be discussed subsequently, this case demonstrates classic cardiac sarcoidosis presenting with decompensated heart failure as well as conduction abnormalities secondary to infiltration of the myocardium and the His-Purkinje system.

## Discussion

Cardiac involvement in sarcoidosis is clinically present in 5% of patients with sarcoidosis, however subclinical involvement based on autopsy is as high as 20 to 40%.^13^-^15^ Cardiac sarcoidosis can occur at any time during the clinical course of sarcoidosis, and can be the presenting feature. The presence of cardiac involvement is associated with a very poor prognosis^8^ and accounts for as many as 13 to 25% of deaths from sarcoidosis.^16^ In an autopsy series, cardiac involvement was seen in 27% of patients whereas clinical involvement was seen in only 18% (history of heart failure, arrhythmia or conduction abnormality).^17^

## Manifestations of cardiac sarcoidosis

Sarcoidosis can affect all tissues and embryological layers of the heart (Table 1). Granulomatous infiltration can affect the conduction system, with complete heart block being the most common presentation in patients with clinical cardiac sarcoidosis (23–30%).^6^ First-degree atrio-ventricular (AV) block and bundle branch blocks can also occur.^6^,^7^ Q waves are most likely indicative of myocardial fibrosis and granulomatous infiltration but rarely, there can be epicardial coronary artery sarcoid involvement, which has even presented as an acute coronary syndrome.^18^ Ventricular arrhythmias are due to active granulomas or myocardial fibrosis (healed granulomas) creating foci of re-entrant activity,^19^ and ventricular tachycardia and pre-ventricular contractions (PVCs) are the second most common presenta-tions of cardiac sarcoidosis (23%).^7^ Sudden death secondary to ventricular fibrillation or complete AV block accounts for 25 to 67% of deaths.^6^,^10^

**Table 1:** COMMONEST CARDIAC MANIFESTATIONS OF SARCOIDOSIS.^6^-^9^,^14^,^40^

*Conduction system*	*%*
AV block	10–30
Frequent PVCs	30
VT	20
SVT	20
Heart failure	
Depressed LVEF	10–70
Valvular insufficiency	20
LV diastolic dysfunction	10–30
Dilated LV	30
LV wall abnormalities	5–25
Sudden cardiac death	30–65

AV = atrioventricular; EF = ejection fraction; LV = left ventricle; PVC = pre-ventricular contraction; SVT = supraventricular tachycardia.

Sarcoid involvement of the myocardium can precipitate heart failure, both systolic and diastolic. Heart failure is the cause of death in 25 to 73% of patients with cardiac sarcoidosis.^6^,^10^ Valvular abnormalities are common due to papillary muscle granulomatous infiltration and fibrosis (particularly the mitral valve), however, granulomatous disease of the valve leaflets themselves has been described.20" Pericardial,^21^,^22^ aortic root^23^,^24^ and even epicardial coronary artery^18^,^25^ sarcoid involvement have all been described in the literature.

Our patient had conduction abnormalities (AV conduction delay, frequent PVCs and sustained ventricular tachycardia) as well as both systolic and diastolic dysfunction and mitral regurgitation secondary to granulomatous infiltration.

## Evaluation of patients with sarcoidosis for cardiac involvement

Numerous modalities exist to evaluate for cardiac involvement of sarcoidosis (Table 2). ECG abnormalities such as those described above or simulated pathological Q waves are present in 70% of patients with sarcoidosis.^9^ Echocardiographic examination can reveal: ventricular aneurysms, valvular insufficiency, and segmental or global hypokinesis.^28^ Radionucleotide examination has also been widely performed.^27^ Areas of decreased thallium-201 uptake correspond to infiltration. However, defects decrease in size during exercise in contrast to ischaemic areas.

PET is more sensitive in detecting early inflammation due to sarcoidosis.^28^ CMR demonstrating late gadolinium enhancement has a sensitivity of 100% and specificity of 78%,^29^ with the uptake seen primarily in the basal and lateral segments and mostly in the mid portion of the myocardium and epicardium. This contrasts with uptake due to infarction, which is primarily in the endocardium. Also commonly seen, again, are wall-motion abnormalities and myocardial thinning. In one study, normalisation of delayed gadolinium enhancement correlated with clinical improvement and clearing of sarcoidosis.^29^

On ECG (Fig. 2), our patient demonstrated not only the conduction abnormalities, but also Q waves inferiorly indicative of an old infarction. CMR (Fig. 3) was also suggestive of multivessel coronary artery disease, however, delayed gadolinium enhancement of the epicardium, myocardium and endocardium was suggestive of myocarditis or infiltrative process. The latter suggestion was confirmed by a normal cardiac catheterisation.

**Table 2:** EVALUATION FOR SARCOID INVOLVEMENT OF THE HEART

*Modality*	*Potential findings*
ECG	Atrioventricular conduction abnormalities, PVCs, VT, Q waves
Echocardiogram	Segmental or global hypokinesis, systolic or diastolic dysfunction, ventricular aneurysms, abnormal wall thickness, valvular insufficiencies
Myocardial perfusion	Decreased uptake of thallium-201 or technicium-99, which improves during exercise
CMR	Late gadolinium enhancement of basal and lateral free-wall myocardium and epicardium, regional or segmental wall-motion abnormalities, focal abnormalities in wall thickness
PET	Increased uptake of F-FDG corresponds to areas of infiltration which parallels peri-hilar uptake

CMR = cardiac magnetic resonance imaging; F-FDG = F-fluorodeoxyglucose; PET = positron emission tomography; PVCs = preventricular contractions; VT = ventricular tachycardia.

## Diagnosis

Granulomas of the pericardium and endocardium have been reported, but the myocardium is more frequently infiltrated. Most commonly involved are the left ventricular free wall and papillary muscles, followed by the basal aspect of the ventricular septum.^6^

The gold standard of sarcoid diagnosis is tissue biopsy, although this has been debated.^30^ Endomyocardial biopsy (EMB) carries a high false-negative rate due to sampling error, as granulomatous infiltration is non-homogenous and more often more basilar, however biopsies are usually taken from the apex and septum. Furthermore, EMB is most often obtained from the right ventricle whereas left ventricular involvement is more common in sarcoidosis. Endomyocardial biopsy guided by findings on CMR has been used in patients with elevated troponin-I and absence of significant obstructive coronary disease, and it improved the diagnostic yield of either modality singly (most common diagnosis was myocarditis).^31^

The Japanese Ministry of Health and Welfare guidelines for the diagnosis of cardiac sarcoidosis are based on either pathological confirmation or a constellation of clinical findings. The histological diagnosis requires the presence of non-caseating granulomas, whereas the clinical diagnosis requires the presence of conduction abnormalities or ventricular arrhythmias plus at least one of the following: abnormal wall motion, wall thickening, left ventricular dilatation; perfusion defect; heart failure; or interstitial fibrosis or more than moderate cellular infiltration on histological examination.^16^

Although EMB was negative for granulomatous disease in our patient, his physical examination and imaging findings certainly still suggested cardiac sarcoidosis, and lung biopsy revealed pathognomonic non-caseating granulomas. As this case illustrates, reliance solely on cardiac biopsy is very low yield and diagnosis of cardiac sarcoidosis requires appreciation of the overall clinical picture.

## Treatment

The mainstay of cardiac sarcoidosis treatment is corticosteroids, although most supporting data are anecdotal and studies that have been performed are retrospective.^9^,^10^ Treatment with methotrexate, azathioprine, chloroquine, cyclophosphamide and infliximab has been described in the literature for patients intolerant of corticosteroids or with progression of cardiac dysfunction despite corticosteroids.^9^,^32^-^34^

Medical therapy for heart failure and pacemakers/implantable cardiodefibrillators are indicated according to current guidelines. Radiofrequency ablation has been successful in treating ventricular tachycardia (VT) refractory to medical therapy.^35^ Transplant is the ultimate therapy in end-stage heart failure or intractable ventricular arrhythmias refractory to both medical therapy and devices. In one series of 65 patients with cardiac sarcoidosis who underwent heart transplant, one-year survival was better than for other transplant indications (87.7 vs 84.5%, p = 0.030). The five-year survival was estimated at 80%.^11^ The incidence of recurrence of cardiac sarcoidosis post-transplant is unknown, however, it has been described in the literature.^36^ Transmission of sarcoidosis has been hypothesised based on case reports (‘donoracquired sarcoidosis’),^37^ and has been described in a patient post heart transplantation.^38^

## Prognosis

Originally, survival after onset of cardiac manifestations was roughly two years based on autopsy data,6 however, more recent studies in patients receiving treatment have the five-year survival at 40 to 60%.^10^,^39^ The cause of death in cardiac sarcoidosis is, again, mostly secondary heart block or ventricular fibrillation. Concomitant pulmonary and cardiac involvement carries a worse prognosis.^8^ A multivariate analysis by Yazaki et al. identified NYHA class, left ventricular end-diastolic diameter, and a history of sustained ventricular tachycardia as independent predictors of mortality.^10^

## Conclusion

Subclinical cardiac involvement in patients with sarcoidosis is common. ECG abnormalities are the commonest presentation of cardiac sarcoidosis. Abnormalities can also be detected on echocardiogram, myocardial perfusion scans, cardiac MRI and PET. Screening of patients with sarcoidosis for cardiac involvement with CMR or PET, especially with abnormal ECG or echocardiograms, should be performed. Cardiac dysfunction secondary to sarcoid infiltration responds to steroid therapy and transplant is an option in patients with severe, symptomatic cardiomyopathy. Patients should also be evaluated for implantable defibrillator, certainly in those with low ejection fraction or a history of ventricular arrhythmia or sudden cardiac death. Lastly, although the five-year survival of patients with cardiac sarcoidosis is roughly 50% with steroid treatment, aggressive medical management of independent predictors of mortality, namely signs and symptoms of heart failure and ventricular tachycardia, may also improve survival.
